# Multi-gene panel testing and association analysis in Cypriot breast cancer cases and controls

**DOI:** 10.3389/fgene.2023.1248492

**Published:** 2023-09-18

**Authors:** Maria Zanti, Maria A. Loizidou, Denise G. O’Mahony, Leila Dorling, Joe Dennis, Peter Devilee, Douglas F. Easton, Mihalis I. Panayiotidis, Andreas Hadjisavvas, Kyriaki Michailidou

**Affiliations:** ^1^ Biostatistics Unit, The Cyprus Institute of Neurology and Genetics, Nicosia, Cyprus; ^2^ Department of Cancer Genetics, Therapeutics and Ultrastructural Pathology, The Cyprus Institute of Neurology and Genetics, Nicosia, Cyprus; ^3^ Department of Public Health and Primary Care, Centre for Cancer Genetic Epidemiology, University of Cambridge, Cambridge, United Kingdom; ^4^ Department of Human Genetics, Leiden University Medical Center, Leiden, Netherlands; ^5^ Department of Pathology, Leiden University Medical Center, Leiden, Netherlands; ^6^ Department of Oncology, Centre for Cancer Genetic Epidemiology, University of Cambridge, Cambridge, United Kingdom

**Keywords:** breast cancer susceptibility, panel sequencing, MASTOS study, case-control, next-generation sequencing

## Abstract

**Introduction:** It is estimated that around 5% of breast cancer cases carry pathogenic variants in established breast cancer susceptibility genes. However, the underlying prevalence and gene-specific population risk estimates in Cyprus are currently unknown.

**Methods:** We performed sequencing on a population-based case-control study of 990 breast cancer cases and 1094 controls from Cyprus using the BRIDGES sequencing panel. Analyses were conducted separately for protein-truncating and rare missense variants.

**Results:** Protein-truncating variants in established breast cancer susceptibility genes were detected in 3.54% of cases and 0.37% of controls. Protein-truncating variants in *BRCA2* and *ATM* were associated with a high risk of breast cancer, whereas PTVs in *BRCA1* and *PALB2* were associated with a high risk of estrogen receptor (ER)-negative disease. Among participants with a family history of breast cancer, PTVs in *ATM*, *BRCA2*, *BRCA1*, *PALB2* and *RAD50* were associated with an increased risk of breast cancer. Furthermore, an additional 19.70% of cases and 17.18% of controls had at least one rare missense variant in established breast cancer susceptibility genes. For *BRCA1* and *PALB2*, rare missense variants were associated with an increased risk of overall and triple-negative breast cancer, respectively. Rare missense variants in *BRCA1*, *ATM*, *CHEK2* and *PALB2* domains, were associated with increased risk of disease subtypes.

**Conclusion:** This study provides population-based prevalence and gene-specific risk estimates for protein-truncating and rare missense variants. These results may have important clinical implications for women who undergo genetic testing and be pivotal for a substantial proportion of breast cancer patients in Cyprus.

## 1 Introduction

Germline pathogenic variants (PVs) in cancer susceptibility genes have been associated with a significant risk of breast cancer (Dorling et al., 2021). Following the National Comprehensive Cancer Network (NCCN) guidelines, medical management recommendations are provided for certain genes contributing to increased risk of breast cancer (Daly et al., 2021). Genetic testing allows patients carrying PVs, to benefit from risk-reducing strategies including closer surveillance at an early age, prophylactic surgeries and chemoprevention, as well as targeted therapies (Pilie et al., 2019).

Gene panel testing of large population-based case-control studies has recently provided improved estimates of the prevalence of PVs and the respective magnitude of breast cancer risk associated with these PVs (Dorling et al., 2021; Hu et al., 2021). It is estimated that around 5% of breast cancer cases harbor PVs in established breast cancer susceptibility genes (*ATM*, *BARD1*, *BRCA1*, *BRCA2*, *CHEK2*, *PALB2*, *RAD51C*, *RAD51D*, *TP53*), of which the most prevalent occur in *BRCA2*, *CHEK2* and *BRCA1* (Dorling et al., 2021; Hu et al., 2021). Recently, it has been reported that 13% of high-risk Cypriot breast cancer patients are positive for PVs in *BRCA1* and *BRCA2* (Loizidou et al., 2017). However, the aggregate prevalence of cancer susceptibility genes among breast cancer cases or controls unselected for family history or age at diagnosis is not yet determined.

Here we used panel sequencing data generated as part of the Breast Cancer Association Consortium (BCAC) BRIDGES project (Dorling et al., 2021), to investigate the prevalence of PVs and rare missense variants in samples from the MASTOS study (Loizidou et al., 2008; Hadjisavvas et al., 2010), a population-based case-control study of breast cancer in Cyprus. We also estimated the risks of breast cancer associated with protein-truncating and rare missense variants in the Cypriot population.

## 2 Materials and methods

### 2.1 Study population

This study included case-control samples from the MASTOS study, a population-based case-control study of breast cancer in Cyprus (Loizidou et al., 2008; Hadjisavvas et al., 2010). The study was approved by the National Bioethics Committee of Cyprus, and all participants provided signed informed consent (Approval No, ΕΕΒΚ/ΕΠ/2016/38) (Loizidou et al., 2008). The study includes 990 breast cancer cases and 1,094 age-matched healthy controls. All study participants were over 18 years of age. The average age of diagnosis for cases was 51.5 ± 9.3 standard deviation (sd) years, while the average age at the time of study enrolment for age-matched controls, was 55.7 ± 6.9 sd. Positive family history of breast cancer was reported for 16.2% (*n* = 158/973) and 8.0% (*n* = 87/1,091) for cases and controls, respectively. The majority of tumors were invasive carcinomas (92.5%, *n* = 656/709), estrogen receptor (ER)-positive (75.8%, *n* = 476/628), progesterone receptor (PR)-positive (61.6%, *n* = 381/619) and human epidermal growth factor receptor 2 (HER2)-negative (83.9%, *n* = 490/584). Out of the 990 tumors, 576 (58.2%) had available data for all ER, PR and HER2, of which 11.5% (*n* = 66/576) had a triple-negative phenotype, 76.6% (*n* = 441/576) had an ER + phenotype, 62.8% (*n* = 362/576) had a PR + phenotype and 16.1% (*n* = 93/576) had a HER2+ phenotype. All study sample characteristics are summarized in Table 1.

**TABLE 1:** Study population characteristics.

Characteristics	Cases (N = 990)	Controls (N = 1,094)
Age^a^		
Mean ± sd—years	51.5 ± 9.3	55.7 ± 6.9
Range—years	26–74	28–71
Age Distribution—N/total N. (%)		
≤29	5/990 (0.5%)	2/1,091 (0.2%)
30–39	87/990 (8.8%)	8/1,091 (0.7%)
40–49	349/990 (35.3%)	178/1,091 (16.3%)
50–59	344/990 (34.7%)	566/1,091 (51.9%)
≥60	205/990 (20.7%)	337/1,091 (30.9%)
Family history of breast cancer^b^—N/total N. (%)		
No	815/973 (83.8%)	1,004/1,091 (92.0%)
Yes	158/973 (16.2%)	87/1,091 (8.0%)
Histological subtype—N/total N. (%)		
Invasive	656/709 (92.5%)	NA
*In situ*	53/709 (7.5%)	NA
ER status—N/total N. (%)		
ER-negative	152/628 (24.2%)	NA
ER-positive	476/628 (75.8%)	NA
PR status—N/total N. (%)		
PR-negative	238/619 (38.4%)	NA
PR-positive	381/619 (61.6%)	NA
HER2 status—N/total N. (%)		
HER2-negative	490/584 (83.9%)	NA
HER2-positive	94/584 (16.1%)	NA
Breast cancer subtypes^c^– N/total N. (%)		
Triple-negative	66/576 (11.5%)	NA
ER-positive	441/576 (76.6%)	NA
PR-positive	362/576 (62.8%)	NA
HER2-positive	93/576 (16.1%)	NA

a Age is denoted as the age at diagnosis for cases or age at interview for controls.

b family history was restricted to first-degree relatives.

c breast cancer subtypes for tumors that have data available for all ER, PR, and HER2 biomarkers.

Estrogen receptor; HER2, human epidermal growth factor receptor 2; NA, non-applicable; PR, progesterone receptor; sd, standard deviation.

### 2.2 Sequence analysis

We analyzed targeted panel sequencing data on 35 actionable and suspected breast cancer susceptibility genes (Supplementary Table S1) using the BRIDGES panel (Dorling et al., 2021). Results are presented for variants in 34 genes since *PPM1D* was shown to be associated with breast or ovarian cancer risk, but in low allelic fractions (“somatic mosaicism”) (Ruark et al., 2013). These are potentially due to treatment, thus excluded from further analysis. Details on library preparation, sequencing and bioinformatics analysis including variant calling and quality control were previously documented (Dorling et al., 2021). In-frame insertions/deletions, intronic variants and variants in untranslated regions (UTRs) were not considered in the analysis.

Protein-truncating variants (PTVs) were defined as frameshift insertions/deletions, splicing variants (±2 positions) and nonsense variants as annotated by ANNOVAR (Wang et al., 2010). Splice variants affecting the penultimate exon (except variants in *ATM*, *BARD1*, *BRCA1*, *RAD51C*, *RAD51C* and *PALB2* for which the truncated protein might still be pathogenic, irrespective of exon skipping; evidence previously documented (Dorling et al., 2021)), as well as PTVs in the last exon of each gene, were excluded from the analysis (Dorling et al., 2021). In addition, six canonical splice variants in *BRCA1* (c.594-2A>C, c.4096 + 1G>A, c.4096 + 2T>C, c.4186-2A>G, c.4358-1G>C and c.4358-2del) were excluded, since they are of uncertain clinical significance according to ENIGMA (Evidence-based Network for the Interpretation of Germline Mutant Alleles) classification schemes (Spurdle et al., 2012).

Classifications for rare missense variants (allele frequency of less than 0.001 in gnomAD v2.1.1 non-Finnish European exome samples) were retrieved from ClinVar (https://www.ncbi.nlm.nih.gov/clinvar/, last accessed 11/12/2022), or the ENIGMA *BRCA1* and *BRCA2* expert panel (https://enigmaconsortium.org/) (Spurdle et al., 2012), along with missense *TP53* variant classification based on ACMG/AMP guidelines (Richards et al., 2015), augmented with classifications made using a quantitative classification model which utilizes bioinformatics prediction tools alongside germline to somatic frequency ratios (Fortuno et al., 2019; Fortuno et al., 2021). Rare missense variants were further annotated for functional protein domain location defined by the UniProt database (https://www.uniprot.org/, release 2022_04). Rare missense variants classified as (likely) benign were not considered in the analysis.

### 2.3 Statistical analysis

Prevalence of protein-truncating and rare missense variants in each gene was tabulated for breast cancer patients and controls. Odds ratios (ORs) with 95% CIs were estimated using Firth’s bias-reduced penalized-likelihood logistic regression for overall, ER-positive and ER-negative breast cancer. For ER-negative cases, we also evaluated the associations for triple-negative breast cancer. Associations were adjusted for age at diagnosis or interview and first-degree family history of breast cancer. Logistic regression *p* values were estimated using Wald’s test. *p* values smaller than 0.05 were considered to be statistically significant. Association analysis was performed separately for PTVs and rare missense variants. Associations for rare missense variants were also evaluated according to gene and gene domain for the established breast cancer susceptibility genes (Dorling et al., 2021). Heterozygous and homozygous carriers of variants in a gene were not distinguished as it was not always possible to do so with certainty, and the number of homozygotes was too small for separate analysis.

Cumulative risks of breast cancer in the absence of other events were calculated by combining age-specific odds ratios with population incidence rates for Cyprus (2020) as a baseline, as previously described (Schmidt et al., 2016).

## 3 Results

All the protein-truncating and rare missense variants are provided in Supplementary Table S2.

### 3.1 Protein-truncating variants

Among 990 breast cancer cases and 1,094 controls, we identified 27 unique PTVs in 4.55% (n = 45/990) of cases and 1.46% (*n* = 16/1,094) of controls (Table 2, Supplementary Table S2). Of these, 3.54% of cases (*n* = 35/990) and 0.37% of controls (*n* = 4/1,094), harbored PTVs in the established breast cancer susceptibility genes *ATM*, *BARD1*, *BRCA1*, *BRCA2*, *CHEK2*, *PALB2*, *RAD51C*, *RAD51D* and *TP53* (Dorling et al., 2021).

**TABLE 2:** Prevalence of protein-truncating variants and associations with overall breast cancer risk.

Gene	Prevalence of PTVs			Adjusted OR (95% CI)	*p*-Value
	Variants	Carriers (N, %)			
		Cases (N = 990)	Controls (N = 1,094)		
***Established breast cancer susceptibility genes***					
***ATM***	**7**	**9 (0.91)**	**1 (0.09)**	**7.61 (1.32–43.97)**	**0.023**
*BRCA1*	1	3 (0.30)	1 (0.09)	3.18 (0.44–23.1)	0.25
***BRCA2***	**6**	**18 (1.82)**	**1 (0.09)**	**9.75 (1.81–52.69)**	**0.0081**
*CHEK2*	1	-	1 (0.09)	0.35 (0.01–8.69)	0.52
*PALB2*	1	5 (0.51)	-	16.14 (0.85–305.14)	0.0637
***Other genes***					
*CDH1*	1	-	1 (0.09)	0.31 (0.01–7.67)	0.48
*ABRAXAS1*	1	1 (0.10)	-	1.61 (0.07–39.77)	0.77
*FANCC*	2	3 (0.30)	2 (0.18)	1.06 (0.2–5.5)	0.94
*FANCM*	1	-	1 (0.09)	0.2 (0.01–4.97)	0.33
*GEN1*	1	-	1 (0.09)	0.2 (0.01–4.97)	0.33
*MRE11*	1	-	1 (0.09)	0.62 (0.03–15.18)	0.77
*MUTYH*	2	1 (0.10)	1 (0.09)	1.2 (0.12–11.77)	0.87
*RAD50*	1	4 (0.40)	1 (0.09)	3.83 (0.56–26.12)	0.17
*RECQL*	2	1 (0.10)	3 (0.27)	0.62 (0.08–4.71)	0.65
*RINT1*	1	-	1 (0.09)	0.26 (0.01–6.37)	0.41
Total	29	46 (4.65)	16 (1.46)		

For the established breast cancer susceptibility genes (Dorling et al., 2021) and other genes included in the panel, odds ratios (ORs) with 95% confidence intervals (CIs) were estimated by adjusting for age and family history of breast cancer (first-degree relatives) using the Firth’s bias-reduced penalized-likelihood logistic regression. *p* values were estimated using the Wald’s test. Significant risks (*p*-value < 0.05, OR >1.0, CI, not including 1) of breast cancer overall, are indicated in bold.

CI, confidence interval; OR, odds ratio; PTVs, protein-truncating variants.

Among the case patients, the highest prevalence of PTVs was observed for *BRCA2* (*n* = 18/990, 1.82%), *ATM* (*n* = 9/990, 0.91%), *PALB2* (*n* = 5/990, 0.51%), *RAD50* (*n* = 4/990, 0.40%), *BRCA1* (*n* = 3/990, 0.3%) and *FANCC* (*n* = 3/990, 0.3%). We also identified one PTV in each of *RECQL*, *ABRAXAS1* and *MUTYH*. Among controls, the highest prevalence was observed for *RECQL* (*n* = 3/1,094, 0.27%) and *FANCC* (*n* = 2/1,094, 0.18%). We also detected one PTV in each of *ATM*, *BRCA1*, *BRCA2*, *CDH1*, *CHEK2*, *FANCM*, *GEN1*, *MRE11*, *MUTYH*, *RAD50* and *RINT1* (Table 2). The founder *BRCA2* c.8756delG PTV was detected in 1.01% of breast cancer cases (*n* = 10/990) and 0.09% of controls (*n* = 1/1,094) which corresponds to more than half of the *BRCA2* PTVs observed among cases (55.56%, *n* = 10/18) and the only *BRCA2* PTV identified among controls.

**FIGURE 1:**
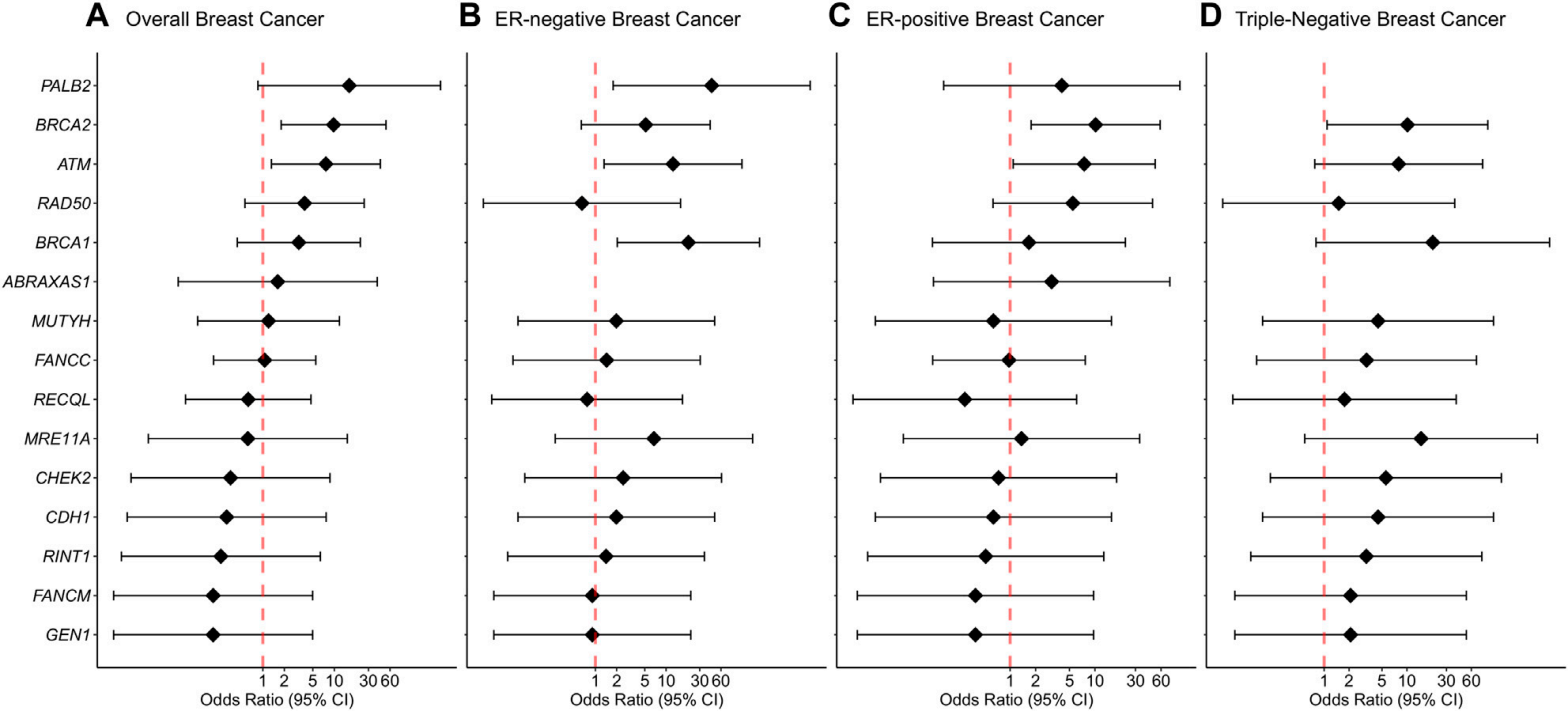
Associations of protein-truncating variants with breast cancer risk. **(A)** Overall breast cancer, **(B)** ER-negative breast cancer, **(C)** ER-positive breast cancer, **(D)** Triple-negative breast cancer. Odds ratios (ORs) and 95% confidence intervals (CIs) for overall breast cancer are represented only for genes with at least one protein-truncating variant detected in either cases or controls. Logistic regression analysis was conducted by adjusting for age and family history of breast cancer (first-degree relatives). The genes are listed in order of decreasing estimated odds ratios for overall breast cancer risk.

We report evidence of PTV association with overall breast cancer risk for *BRCA2* and *ATM* with adjusted ORs of 9.75 (1.81-52.69 95% CI, *p*-value = 0.0081) and 7.61 (1.32-43.97 95% CI, *p*-value = 0.023) (Figure 1; Table 2, Supplementary Table S3). Association with overall breast cancer is also depicted for PTVs in *PALB2* with adjusted ORs of 16.14 (0.85-305.14 95% CI, *p*-value = 0.064), although not statistically significant (Figure 1; Table 2, Supplementary Table S3)*.* For other genes included in the panel, the evidence for an association between overall breast cancer and PTVs did not reach statistical significance (Figure 1; Table 2, Supplementary Table S3).

Breast cancer cases with PTVs in *BRCA1* and *BRCA2* had a relatively younger age at diagnosis (46.7 ± 10.4 sd) compared to cases without PTVs in *BRCA1* and *BRCA2* (51.6 ± 9.3 sd, Wilcoxon rank sum test *p*-value = 0.046) or cases with PTVs in genes other than *BRCA1* and *BRCA2* (53 ± 10.9 sd, two-sample *t*-test *p*-value = 0.051). The associations with breast cancer overall for both participants diagnosed or recruited at an age earlier or later than 50 years did not reach statistical significance, except for *BRCA2* PTV-carriers who were at an increased risk of the disease if diagnosed at an age later than 50 years (Supplementary Table S4). Analysis by family history of breast cancer (first-degree relatives) revealed that the prevalence of PTVs among patients with a family history of breast cancer (8.23%, *n* = 13/158) was about two times the frequency reported among patients with no family history of breast cancer (3.56%, *n* = 29/815; *p*-value = 0.015). To investigate the influence of a family history of breast cancer, analysis was conducted separately for cases with and without a first-degree relative with breast cancer. Among participants with family history of breast cancer, PTVs in *ATM* (*p*-value = 0.002), *BRCA2* (*p*-value = 0.005), *BRCA1* (*p*-value = 0.046), *PALB2* (*p*-value = 0.039) and *RAD50* (*p*-value = 0.044) were significantly associated with increased risk of breast cancer. The exclusion of breast cancer patients with a family history of breast cancer had a minor impact on PTV association with overall breast cancer risk (i.e., *BRCA2* adjusted OR of 8.74; 1.57-48.75 95% CI, *p*-value = 0.013) (Supplementary Table S5).

Protein-truncating variants in *BRCA2* were strongly associated with an increased risk of ER-positive and triple-negative breast cancer with adjusted ORs of 10.18 (1.77-58.55 95% CI, *p*-value = 0.009) and 10.14 (1.08-94.84 95% CI, *p*-value = 0.042), respectively. Protein-truncating variants in *BRCA1* were strongly associated with an increased risk of ER-negative breast cancer with adjusted ORs of 20.73 (2.04-210.69 95% CI, *p*-value = 0.0104). Protein-truncating variants in *ATM* were associated with an increased risk of both ER-negative and ER-positive disease with adjusted ORs of 12.55 (1.33-118.68 95% CI, *p*-value = 0.027) and 7.47 (1.09-51.23 95% CI, *p*-value = 0.041), respectively. Finally, PTVs in the *PALB2* gene were associated with an increased risk of ER-negative breast cancer with adjusted ORs of 44.23 (1.78-1098.24 95% CI, *p*-value = 0.021) (Supplementary Table S3).

**FIGURE 2:**
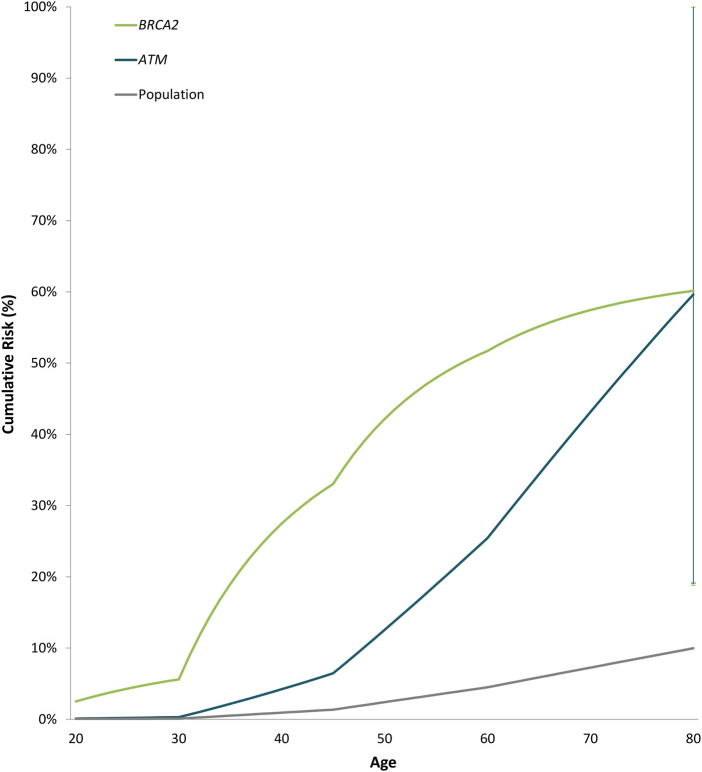
Estimated cumulative risk of breast cancer associated with protein-truncating variants in genes with significant evidence of an association with breast cancer overall. Cumulative risks of breast cancer in the absence of other events were calculated by combining age-specific odds ratios with population incidence rates for Cyprus (2020) as a baseline. The error bars indicate 95% confidence intervals.

Odds ratios decreased significantly with increasing age for *BRCA2* with ORs of 0.94 (0.89-0.99 95% CI, *p*-value = 0.013) (Supplementary Table S6). Estimated cumulative risks of breast cancer in the absence of other events were calculated by combining age-specific odds ratios with population incidence rates for Cyprus (2020) (Figure 2). For carriers of PTVs in *BRCA2* and *ATM* the estimated cumulative risks by 80 years of age exceeded the 30% threshold for high risk, as defined by the NICE (National Institute for Health and Care Excellence) surveillance screening guidelines (NICE, 2019).

### 3.2 Rare missense variants

Among 990 breast cancer cases and 1,094 controls, we identified 380 unique rare missense variants in 50.40% of cases (*n* = 499/990) and 41.77% of controls (*n* = 457/1,094) (Supplementary Table S2). Of these, 147 unique rare missense variants were detected in the established breast cancer susceptibility genes, among 195 out of 990 cases (19.70%) and 188 out of 1,094 controls (17.18%). None of the detected rare missense variants were classified as pathogenic.

**FIGURE 3:**
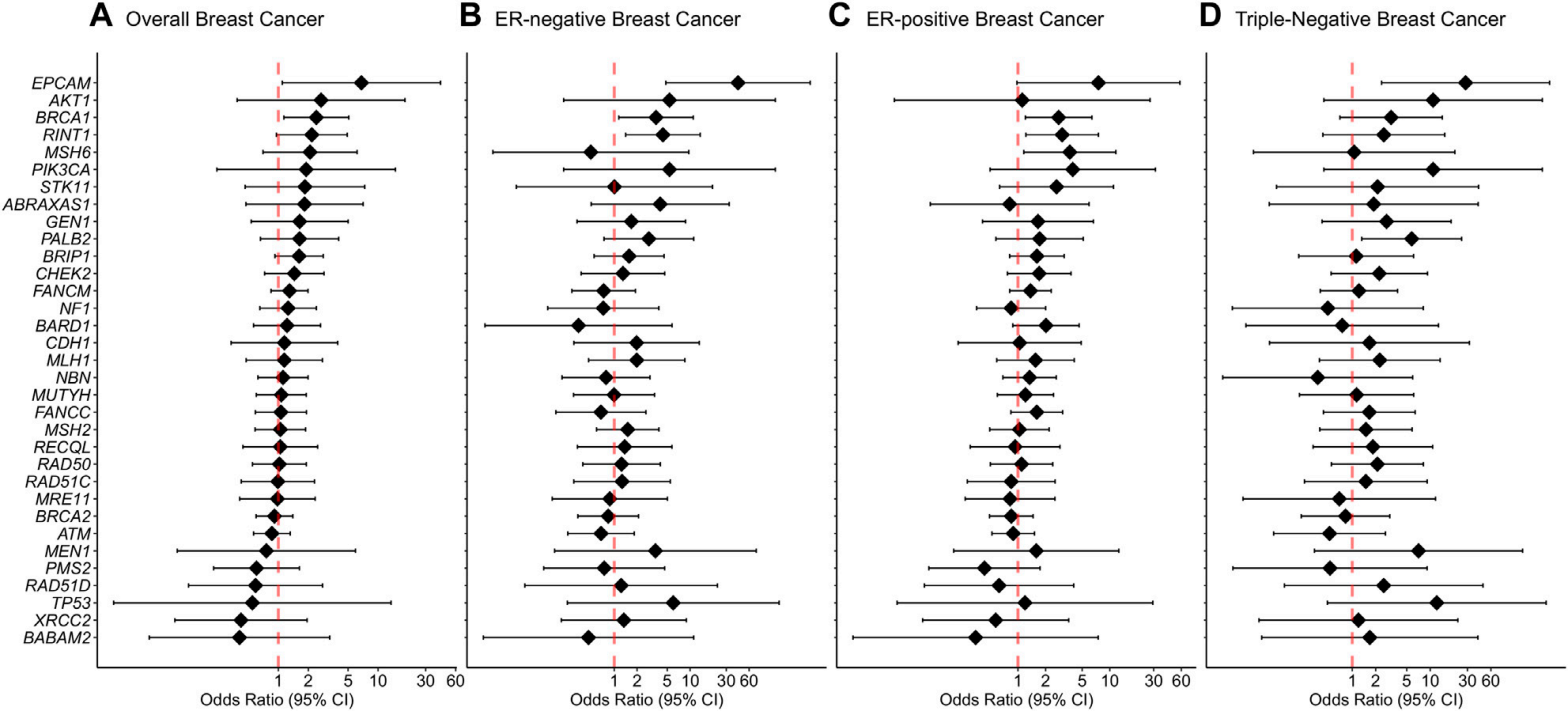
Associations of rare missense variants with breast cancer risk. **(A)** Overall breast cancer, **(B)** ER-negative breast cancer, **(C)** ER-positive breast cancer, **(D)** Triple-negative breast cancer. Odds ratios (ORs) and 95% confidence intervals (CIs) for overall breast cancer are represented only for genes with at least one missense variant detected in either cases or controls. Logistic regression analysis was conducted by adjusting for age and family history of breast cancer (first-degree relatives). The genes are listed in order of decreasing estimated odds ratios for overall breast cancer risk.

Rare missense variants in *BRCA1* were associated with an increased risk of overall breast cancer with an adjusted OR of 2.4 (1.14-5.08 95% CI, *p*-value = 0.022) (Figure 3). In the subtype-stratified analyses, rare missense variants in *BRCA1* were associated with an increased risk of both ER-negative and ER-positive disease with adjusted ORs of 3.56 (1.15-11.01 95% CI, *p*-value = 0.028) and 2.77 (1.21-6.38 95% CI, *p*-value = 0.016), respectively (Supplementary Table S7, Figure 3). Increased risk of triple-negative breast cancer was observed for carriers of rare missense variants in *PALB2* with an adjusted OR of 5.79 (1.32-25.29 95% CI, *p*-value = 0.020, Figure 3).

For missense variants in aggregate, the evidence for an association between overall breast cancer and any protein domain did not reach statistical significance. However, there was a significantly increased risk of ER-negative breast cancer associated with variants located within the *BRCA1* Zinc-Finger, *ATM* FAT, *PALB2* WD1 and *CHEK2* FHA domains. In addition, rare missense variants within the *ATM* PI3L/PI4K catalytic domain demonstrated an association with an increased risk of ER-positive disease. Furthermore, rare missense variants within *PALB2* domains WD1, domain for stimulation of POLH DNA synthesis and domain for interaction with RAD51, BRCA2 and POLH were significantly associated with increased risk of triple-negative breast cancer. Detailed results from the domain-specific analysis are shown in Supplementary Table S8.

## 4 Discussion

This study evaluates the prevalence of protein-truncating and rare missense variants in putative breast cancer susceptibility genes and estimates population-specific risks of breast cancer in a large case-control dataset of breast cancer in Cyprus. We used panel sequencing data from 990 breast cancer cases unselected for family history of breast and/or ovarian cancer or age at disease diagnosis and 1,094 age-matched controls, generated as part of the Breast Cancer Association Consortium (BCAC) BRIDGES project (Dorling et al., 2021).

It is generally estimated that around 5%–6% of breast cancer cases and 1%–2% of the general population, harbor PVs in established breast cancer susceptibility genes (Dorling et al., 2021; Hu et al., 2021; Southey et al., 2021). Among breast cancer patients the most prevalent occur in *BRCA2*, *CHEK2, BRCA1* and *ATM* (Dorling et al., 2021; Hu et al., 2021). Here, we report that 3.54% of breast cancer cases and 0.37% of controls in Cyprus are positive for PTVs in the established breast cancer susceptibility genes (Dorling et al., 2021). Among patients and the established breast cancer susceptibility genes, PTVs in *BRCA2* and *ATM*, followed by *PALB2*, and *BRCA1*, were the most prevalent.

The prevalence of PVs in established breast cancer susceptibility genes possesses substantial ethnic and geographic disparities (Yadav et al., 2021). In Cyprus, unique founder PVs and more frequent *BRCA2* PVs have been observed compared to other countries (Hadjisavvas et al., 2004; Loizidou et al., 2017). Protein-truncating variants in *BRCA2* were the most prevalent in our study (1.82%), with the founder *BRCA2* c.8756delG PTV being detected in ten breast cancer cases (1.01%) which corresponds to more than half of the *BRCA2* PTVs observed among cases. Furthermore, *BRCA2* PTVs were associated with a high risk of breast cancer overall, ER-positive and triple-negative disease and exceeded the 80-year cumulative risk threshold for high risk (30%), as defined by the NICE surveillance screening guidelines ((NICE) 2019). The cumulative risk estimates for *BRCA2* PTVs in Cyprus are higher compared to risks reported by population- (Dorling et al., 2021; Hu et al., 2021) and family-based studies (Kuchenbaecker et al., 2017). However, it is consistent with cumulative risk estimates published by Kuchenbaecker et al. (2017).

Protein-truncating variants in *BRCA1* generally confer around 8 to 10-fold increased risk of breast cancer (Antoniou et al., 2003; Kuchenbaecker et al., 2017; Dorling et al., 2021; Hu et al., 2021). In our study, *BRCA1* PTVs showed weak associations with overall breast cancer risk (*p* > 0.05). However, *BRCA1* was associated with an increased risk of ER-negative disease and was identified as a high-risk gene among case patients who had a first-degree relative with breast cancer. Moreover, breast cancer patients with PTVs in *BRCA1* and *BRCA2* had a relatively younger age at diagnosis compared to cases without PTVs in *BRCA1* and *BRCA2* or cases with PTVs in genes other than *BRCA1* and *BRCA2*. These findings are consistent with recent data suggesting that the prevalence of PVs in genes other than *BRCA1* and *BRCA2* does not depend on age at diagnosis (Tung et al., 2016; Buys et al., 2017).

Among the established breast cancer susceptibility genes, *ATM* yielded an odds ratio of approximately 8 for breast cancer overall and its estimated cumulative risks by 80 years of age exceeded the 30% threshold for high risk [(NICE) 2019], compared to published moderate-risk estimates for *ATM* PTVs (Dorling et al., 2021). Although, if validated in a larger case-control series of breast cancer in Cyprus, it will be of clinical importance.

Pathogenic variants in *PALB2* were previously identified as high- and moderate-risk in the large-scale population-based BRIDGES (Dorling et al., 2021) and CARRIERS projects (Hu et al., 2021). Here we report a possible association of *PALB2* PTVs with high risk of breast cancer overall (*p* = 0.06) and ER-negative disease (*p* < 0.05). However, among cases with a family history of breast cancer, PTVs in *PALB2* were significantly associated with a high risk of disease (*p* < 0.05), a finding consistent with reported associations (Yang et al., 2020; Hu et al., 2021). Furthermore, according to Cybulski et al. (2015) it is estimated that 34% of breast cancer patients with a germline *PALB2* PV have a triple-negative phenotype. We have recently reported that among 163 *BRCA*-negative triple-negative breast cancer patients in Cyprus, 4.3% are positive for PVs in *PALB2* (Zanti et al., 2020), whereas *PALB2* PVs consisted 87.5% of the PVs detected using a panel of 94 cancer susceptibility genes. In the analysis presented here, triple-negative breast cancer patients did not carry any PTVs in *PALB2*. This may be due to the limited number of triple-negative breast cancer cases in our dataset. Hence, additional studies are required to draw more definite conclusions.

It is estimated that variants of uncertain clinical significance (VUS) account for around 30%–40% of the total number of variants identified in gene-panel sequencing studies (Tung et al., 2016; Buys et al., 2017; Federici and Soddu, 2020). Among all women and genes tested, we report a 45.65% prevalence of unclassified rare missense variants. Of these, 19.70% of breast cancer cases and 17.18% of controls had at least one rare missense variant detected in the established breast cancer susceptibility genes (Dorling et al., 2021). This is consistent with the frequency reported in the large-scale CARRIERS project (Hu et al., 2021). We further demonstrate that rare missense variants in *BRCA1* were associated with an increased risk of breast cancer overall. In addition, rare missense variants in *PALB2* were associated with a moderate risk of triple-negative breast cancer. For missense variants in aggregate, rare missense variants in specific domains in *BRCA1*, *ATM*, *CHEK2,* and *PALB2* were significantly associated with increased risk of certain breast cancer subtypes. However, a possible caveat that should be recognized is the potential presence of missense pathogenic variants at an allele frequency higher than 0.001 which were not included in the current analyses.

Overall, we report that 3.54% of breast cancer cases in Cyprus are positive for PTVs in the established breast cancer susceptibility genes. We further provide population-specific evidence for the association of *BRCA2* and *ATM* PTVs with overall breast cancer risk, and ER-negative breast cancer for *PALB2* PTVs. Among the established breast cancer susceptibility genes, the most prevalent PTVs occurred in the *BRCA2* and *ATM*, followed by *PALB2*, and *BRCA1*. Finally, we confirm the effect of family history, age at diagnosis and tumor subtype as critical factors important for risk stratification of women with breast cancer in the general population of Cyprus. These results, in combination with other risk factors, may have important clinical implications for women who undergo genetic testing for breast cancer susceptibility and be beneficial for a substantial proportion of breast cancer patients in Cyprus.

## Data Availability

Carriers of variants included in the BRIDGES project can be found here: https://databases.lovd.nl/shared/screenings#order=id%2CASC&search_owned_by_=BRIDGES%20consortium&page_size=100&page=1 https://databases.lovd.nl/shared/view/BRCA1#object_id=VariantOnTranscript%2CVariantOnGenome%2CScreening%2CIndividual&id=BRCA1#=VariantOnTranscript%2FDNA%2CASC&search_transcriptid=00003478&search_owned_by_=BRIDGES%20consortium&page_size=100&page=1 https://bcac.ccge.medschl.cam.ac.uk/bcacdata/bridges/. Requests for raw data can be made to the Data Access Coordination Committee (DACC) of BCAC (http://bcac.ccge.medschl.cam.ac.uk/).
